# Characterization of Mammalian Selenoprotein O: A Redox-Active Mitochondrial Protein

**DOI:** 10.1371/journal.pone.0095518

**Published:** 2014-04-21

**Authors:** Seong-Jeong Han, Byung Cheon Lee, Sun Hee Yim, Vadim N. Gladyshev, Seung-Rock Lee

**Affiliations:** 1 Department of Biochemistry, Research Center for Aging and Geriatrics, Research Institute of Medical Sciences, Chonnam National University Medical School, Gwangju, Republic of Korea; 2 School of Biological Sciences and Technology, Chonnam National University, Gwangju, Republic of Korea; 3 Division of Genetics, Department of Medicine, Brigham & Women’s Hospital and Harvard Medical School, Boston, Massachusetts, United States of America; University of Wisconsin - Madison, United States of America

## Abstract

Selenoproteins exhibit diverse biological functions, most of which are associated with redox control. However, the functions of approximately half of mammalian selenoproteins are not known. One such protein is Selenoprotein O (SelO), the largest mammalian selenoprotein with orthologs found in a wide range of organisms, including bacteria and yeast. Here, we report characterization of mammalian SelO. Expression of this protein could be verified in HEK 293T cells by metabolic labeling of cells with ^75^Se, and it was abolished when selenocysteine was replaced with serine. A CxxU motif was identified in the C-terminal region of SelO. This protein was reversibly oxidized in a time- and concentration-dependent manner in HEK 293T cells when cells were treated with hydrogen peroxide. This treatment led to the formation of a transient 88 kDa SelO-containing complex. The formation of this complex was enhanced by replacing the CxxU motif with SxxC, but abolished when it was replaced with SxxS, suggesting a redox interaction of SelO with another protein through its Sec residue. SelO was localized to mitochondria and expressed across mouse tissues. Its expression was little affected by selenium deficiency, suggesting it has a high priority for selenium supply. Taken together, these results show that SelO is a redox-active mitochondrial selenoprotein.

## Introduction

Selenium is an essential trace element that supports biological functions by being a component of selenocysteine (Sec), the 21^st^ proteinogenic amino acid [1], [2], [3]. The synthesis of Sec-containing proteins (selenoproteins) involves a unique mechanism to co-translationally incorporate Sec into a growing peptide chain. In particular, Sec insertion sequence (SECIS) element in the 3′-untranslated region (3′-UTR) in eukaryotes is necessary for recruitment of tRNA^Sec^ to insert Sec in response to UGA codon [4], [5], [6]. A combination of SECIS elements and in-frame UGA codons has been adopted for *in silico* characterization of selenoproteomes (full sets of selenoproteins) in diverse organisms [6], [7], [8]. Twenty five selenoprotein genes have been identified in humans *via* this *in silico* approach and further verified experimentally [6].

Following identification of selenoproteins, their biological functions have been studied extensively. Interestingly, most functionally characterized selenoproteins are oxidoreductases that participate in various redox processes such as antioxidant defense, redox regulation of biological functions, redox signaling, and many other oxidoreductase reactions [9]–[13]. Sec supports a higher catalytic efficiency than Cys owing to its higher polarizability and lower p*K*a (∼5.2) value, and perhaps due to other properties [14], [15]. Being a superior catalyst, it is not surprising that Sec may replace catalytic Cys in thiol oxidoreductases [16]–[18].

Up to date, functions have been characterized for approximately half of mammalian selenoproteins. SelO is the largest protein among the 25 mammalian selenoproteins and has been hypothesized to have a kinase domain [19], [20]. In the present study, we characterized human SelO with regard to localization, Sec incorporation, redox interactions and regulation by dietary selenium status.

## Materials and Methods

### Cells, Tissues and Transfection

Human embryonic kidney (HEK) 293T cells were maintained with Dulbecco’s modified Eagle’s medium (DMEM) supplemented 10% fetal bovine serum (FBS) or bovine calf serum (BCS; Hyclone, UT, USA) and antibiotics (1% penicillin and streptomycin; Invitrogen, Carlsbad, CA, USA) humidified air containing 5% CO_2_ at 37°C. Cells were transfected with SelO mutant constructs using calcium phosphate transfection methods. Twenty four hours after transfection, cells were washed and treated with hydrogen peroxides (H_2_O_2_) at various concentrations and times indicated.

### Selenium Diet Study

Three-week old C57BL/6 male mice were purchased from the Jackson Laboratory (Bar Harbor, ME). After a week of acclimation, the mice were randomly divided into four groups (n = 5 animals per group) and selenium diets for 8 weeks. These diets were purchased from Harland Teklad (Madison, MI) and varied in selenium levels, i.e., 0, 0.1, 0.4, and 2.25 ppm. Animals were euthanized at the age of 12 weeks and their tissues were harvested and immediately frozen in liquid nitrogen until further use. All animal experiments were approved by the Brigham and Women’s Hospital and Harvard Medical School Institutional Animal Care and Use Committee.

### Specific SelO Antibody

Polyclonal antiserum against human SelO was raised in rabbits. Rabbits were immunized with KLH-conjugated synthetic peptide containing the deduced amino acids C-^642^ADGADGRQRSYSSKPPL^658^-NH_2_. Cysteine was appended at the N-terminus for KLH conjugation. Anti-SelO IgG was purified by affinity chromatography using an affinity matrix linked with the antigen peptide. Both the synthetic peptide and affinity gel matrix column were manufactured by Peptron (Daejeon, Korea). Purified anti-SelO was eluted with 100 mM glycine (pH 2.5) and the antibody fractions were neutralized in 1 M Tris-HCl (pH 8.0) buffer.

### Constructs and Polymerase Chain Reaction (PCR)

Human SelO cDNA was purchased as cDNA clone (IMAGE ID: 6041782) from imaGenes (Berlin, Germany). cDNA was amplified from EconoTaq PLUS GREEN 2X Master Mix (Lucigen, Middleton, WI, USA) with 5% volumes of dimethyl sulfoxide (Sigma, St. Louis, MO, USA). The pCI-Toxo-SECIS vector contains a highly efficient *Toxoplasma gondii* SelT SECIS element [21]. SelO MLS sequence was cloned into the Nhe1/EcoR1 restriction sites of the pCI-SelK-Toxo-SECIS vector modified from the pCI-neo (Promega, Madison, WI, USA) [22]. We cloned the SelO open reading frame (ORF) and then replaced the SelK ORF in XbaI/SalI restriction sites. Primer sequences are shown in the Table S1. Primer pairs 1 and 2 were used and digested with NheI/EcoRI restriction enzymes to prepare the first MLS cloned into pCI SelK-Toxo-SECIS constructs. The forward primer 3 and the reverse 4–9 primers were used and digested with XbaI/SalI to prepare SelO constructs. To investigate the subcellular localization of SelO, we cloned SelO ORF into pEGFPN1 vector with NheI//SalI restriction sites. Sequences of constructs were verified by sequencing at Genewiz (Boston, MA, USA).

### ^75^Se Metabolic Labeling and Identifying Human SelO

HEK293T cells were metabolically labeled with ^75^Se for 40 h. The cells were lysed in 0.1% NP40 cell lysis buffer (20 mM Tris-HCl, pH 7.4, 150 mM NaCl, 5% Glycerol) containing protease inhibitor (Roche Applied Science, Indianapolis, IN, USA) and 20 mM *N*-ethylmaleimide. The cell lysates were sonicated for 15 min and centrifuged at 13,000 rpm for 10 min at 4°C. The supernatants were precleared with 30 µl of 50% slurry of Protein A/G agarose (SantaCruz Biotechnology, CA, USA) for 1 h. The precleared samples were incubated with 10 µl of anti-SelO antibody for 2 h. Then 30 µl of 50% slurry Protein A/G agarose was added and incubated for 2 h at 4°C. The beads were washed five times with lysis buffer (0.1% NP40, 20 mM Tris-HCl, pH 7.4, and 150 mM NaCl). Bound proteins were eluted with 1x LDS sample buffer (Invitrogen) by boiling for 10 min at 70°C. The samples were electrophoresed on 10% Bis-Tris Gels (Invitrogen) and transferred to PVDF membranes. ^75^Se radioactivity was visualized by using a Phosphorimager system (GE Healthcare, Parsippany, NJ, USA).

### Affinity Purification

For affinity purification, HA-TEV tagged SelO and their binding proteins were expressed in HEK293T cells, and lysed in 0.1% NP40 lysis buffer (20 mM Tris-HCl, pH 7.4, 150 mM NaCl, 5% Glycerol) containing a protease inhibitor and 20 mM NEM. The cell lysates were immunoprecipitated with anti-HA monoclonal antibody (Sigma). The beads were washed 5 times with lysis buffer and incubated with AcTEV protease (Invitrogen) in 0.1 ml of lysis buffer at 4°C for overnight. All samples were analyzed by Western blotting.

### Western Blotting

Sodium dodecyl sulfate (SDS) polyacrylamide gel electrophoresis analysis was carried out using NuPAGE Novex gels (Invitrogen), and the proteins were transferred onto PVDF membranes (Invitrogen), followed by blocking with 5% skim milk for 1 h, and an incubation with primary antibodies overnight at 4°C. The membranes were washed with TBST (25 mM Tris, pH 7.4, 150 mM NaCl, 2 mM KCl and 0.01% Tween 20), and incubated with anti-rabbit or mouse IgG horseradish peroxidase-conjugated antibodies (GE Healthcare) for 1 h at room temperature, and then washed with TBST buffer. Immunoreactive proteins were visualized using an enhanced chemiluminescence detection system (Pierce Biotechnology, Rockford, IL, USA).

### Isolation of Mitochondria and SelO Localization

Mitochondria were isolated from the HEK293T cells transfected with wild type (CxxU) SelO constructs. Forty eight hours after transfection, cells were collected and washed two times with PBS, and then homogenized with Dounce homogenizer in extraction buffer (10 mM HEPES, pH 7.5, 200 mM mannitol, 70 mM sucrose, 1 mM EGTA, and 2 mg/ml BSA) containing protease inhibitor (Roche) and 20 mM *N*-ethylmaleimide. The homogenate was centrifuged at 600 g for 10 min and the supernatant was further centrifuged at 11,000 g for 10 min. The supernatant was the cytosolic fraction. The pellet was resuspended in extraction buffer without BSA and centrifuged at 600 g for 10 min. Then, the supernatant was re-centrifuged at 11,000 g for 10 min. Finally, the pellet was the mitochondrial fraction. The isolated fractions were examined for purity by Western blotting using several organelle-specific antibodies. The mitochondrial marker antibody was thioredoxin reductase 2 (TR2; also known as Thioredoxin reductase TR3) and cytosolic marker antibodies were thioredoxin reductase 1 (TR1) and tubulin antibodies.

The SelO ORF was cloned into pEGFP-N1 vector (Clontech, Palo Alto, CA, USA) to confirm subcellular localization of SelO. The SelO construct was transfected into HEK293T cells. After 48 h, cells were washed with PBS and stained with Mito-Tracker Red (Molecular Probes, Eugene, OR, USA) for 15 min at a final concentration of 50 nM. The cells were washed three times with PBS and fixed with 10% paraformaldehyde for 15 min. After staining, EGFP and Mito-Tracker fluorescence were visualized using a confocal LSM 700 microscope (Carl Zeiss, Oberkochen, Germany).

## Results and Discussion

### SelO Localizes to Mitochondria

**Figure 1:**
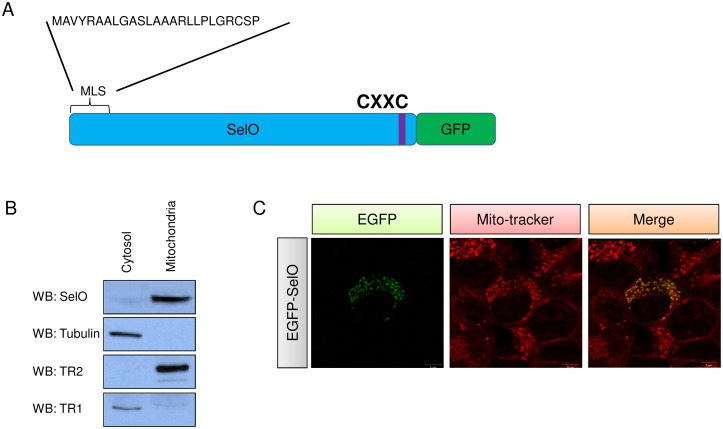
Mitochondrial localization of human SelO. (A) Schematics of recombinant SelO. It contains the mitochondrial leader sequence (MLS) at the N-terminus and the green fluorescent protein (GFP) at the C-terminus. Sec was changed to Cys. (B) Western blotting of SelO, TR2, tubulin, and TR1 in two separate fractions, cytosolic and mitochondrial, isolated from HEK293T cells. (C) Mitochondrial localization of recombinant SelO. The EGFP signal is shown in green and Mito-Tracker in red.

Among the 25 mammalian selenoproteins, only two selenoproteins, TR2 and glutathione peroxidase 4 (GPX4), were shown to reside in mitochondria with function in regulation of mitochondrial redox homeostasis [10], [23]. The presence of a mitochondrial leader sequence (MLS) in SelO (Fig. 1A) suggested it localizes to mitochondria. We prepared cytosolic and mitochondrial fractions of HEK293T cells and examined them by Western blotting (Fig. 1B). We confirmed the purity of cellular fractions by analyzing proteins known to reside in the cytosol and mitochondria. SelO was found exclusively in the mitochondrial fraction, indicating its localization in this compartment. To further verify its mitochondrial localization, we assayed intracellular SelO localization using the recombinant human GFP-fused SelO containing Cys in the place of Sec at its C-terminal end (Fig. 1A). EGFP (Green) and Mito-Tracker (Red) co-localization confirmed that SelO localized to mitochondria (Fig. 1C). Taken together, these results demonstrate that SelO is targeted to mitochondria in mammals.

### Redox Interactions of SelO

**Figure 2:**
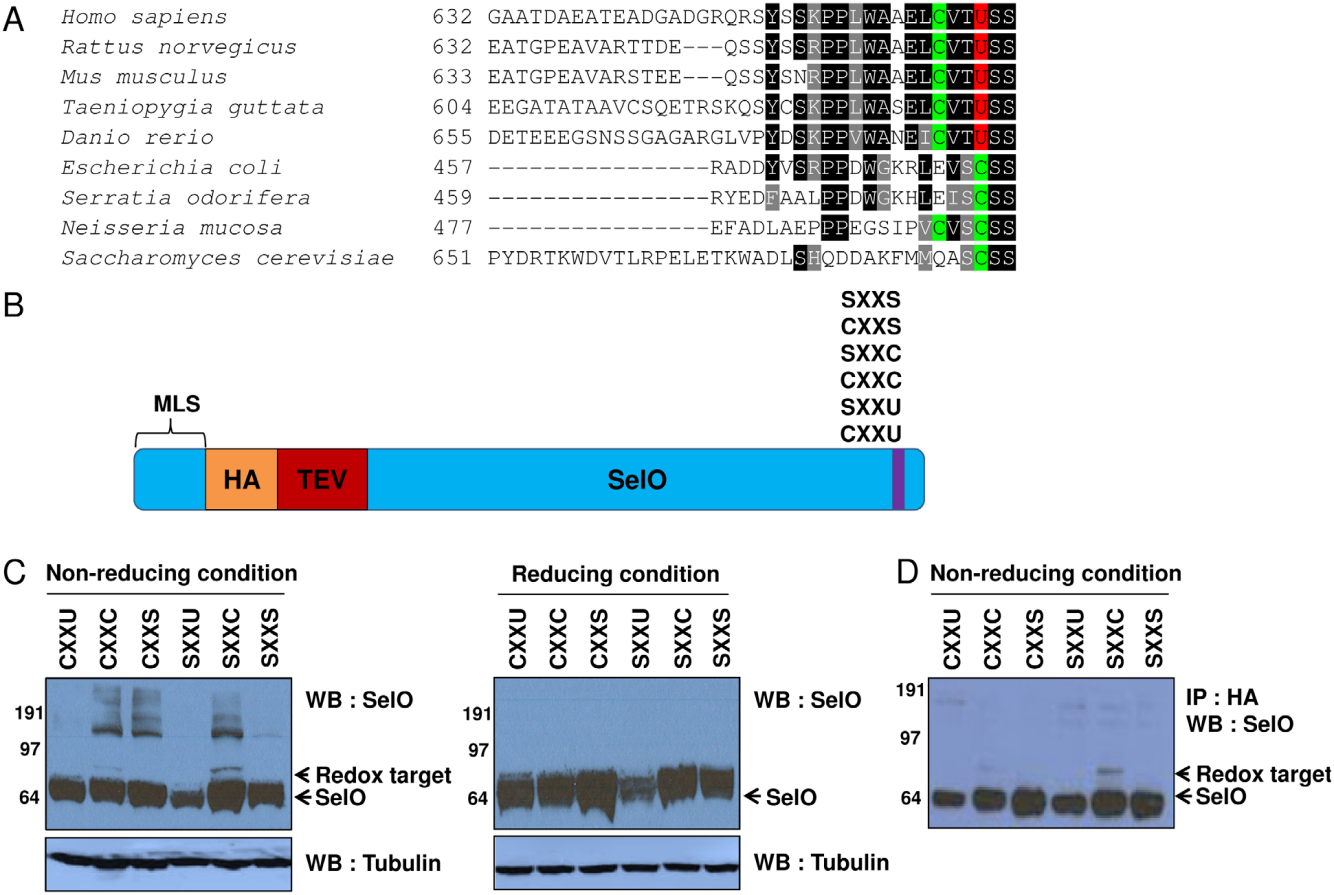
Redox interactions involing human SelO. (A) Protein sequence alignment of the C-terminal region of SelO. (B) Recombinant SelOs. The plasmid construct was designed to trap the interacting partner. The mitochondrial leader sequence (MLS) was flanked by the HA-TEV sequence and the CXXU was changed to SXXU, CXXC, SXXC, CXXS, or SXXS. (C–D) Western blotting of SelO with (C) the cell lysates or (D) the samples that were prepared by using immunoprecipitation with anti-HA antibody from HEK293T cells expressing recombinant human SelO or its mutant forms (CXXU, CXXC, CXXS, SXXU, SXXC, SXXS).

**Figure 3:**
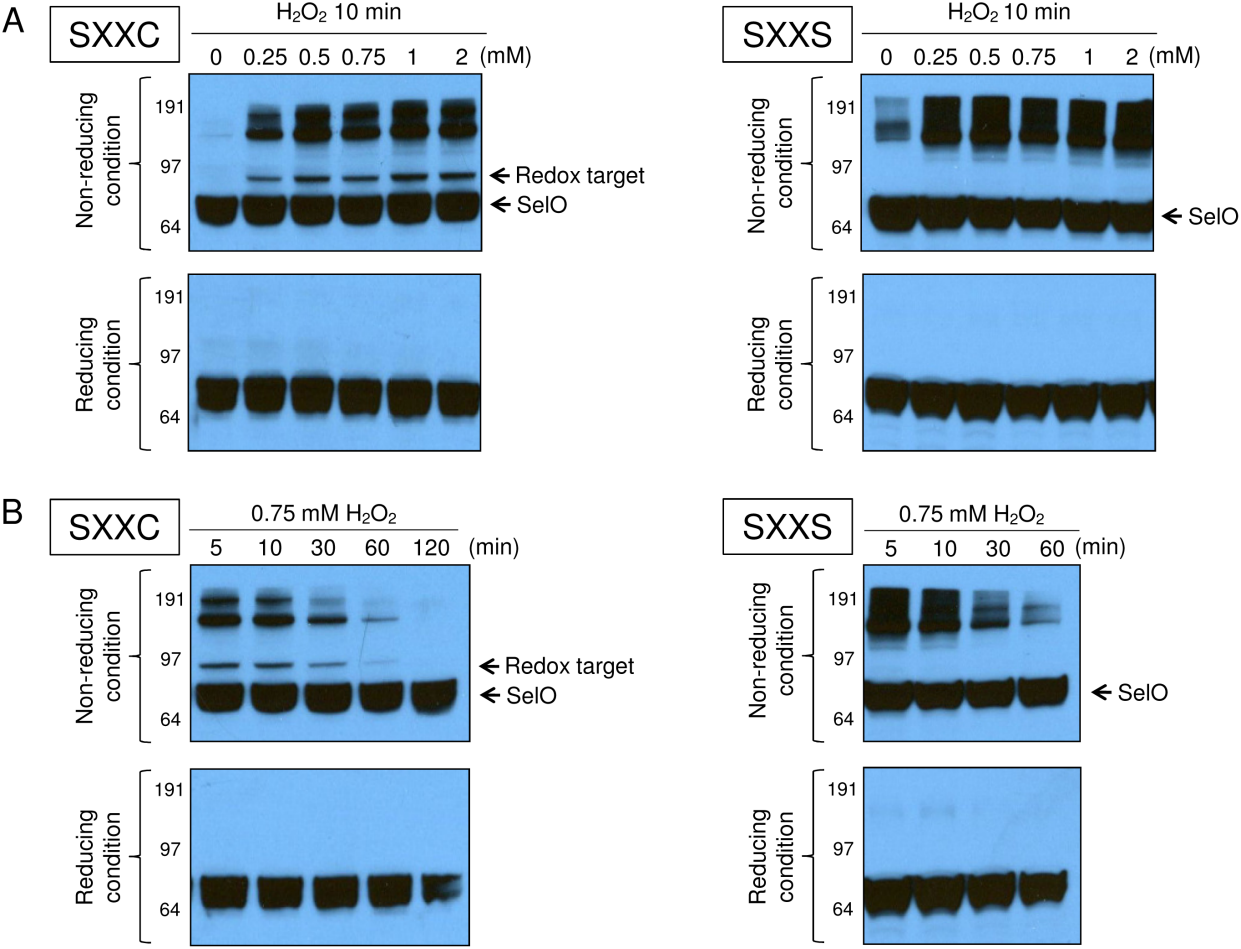
Effect of hydrogen peroxide on redox interactions involving SelO. (A) 0, 0.25, 0.5, 0.75, 1, or 2 mM hydrogen peroxide was added for 5 min to HEK293T cells expressing recombinant human SelO proteins containing either SXXC or SXXS. (B) 0.75 mM hydrogen peroxide was added to HEK293T cells expressing recombinant human SelO proteins containing either SXXC or SXXS motifs for 5, 10, 30, 60, or 120 min. Then, all cell lysates were subjected to Western blotting with anti-SelO antibodies under reducing or non-reducing condition.

Most of the functionally characterized selenoproteins are oxidoreductases that contribute to various redox functions in biological systems [11]. The occurance of the CXXU (with Cys^664^ and Sec^667^ separated by two other residues) motif in human SelO suggested that, like other selenoproteins, SelO may have redox activity. The CXXU motif is conserved across a broad range of organisms in the form of either the CXXU or the CXXC motif (although some of the more distant sequences lack the Cys in the motif), suggesting that Sec and Cys in the CXXU motif may act as catalytic and resolving residues (Fig. 2A). Identifying a redox target protein interacting with the conserved CXXU motif can be crucial in understanding the role of SelO as a redox-active protein To test this, we prepared recombinant human SelO in which the CXXU motif was mutated to CXXC, CXXS, SXXU, SXXC, or SXXS and a HA-TEV tag was flanked by upstream MLS and itself upstream of SelO (Fig. 2B). These recombinant proteins were expressed in HEK293T cells, which were treated with H_2_O_2_, and the cell lysates were prepared for Western blotting or subjected to immunoprecipitation with HA-specific antibodies. We then liberated SelO by using TEV protease-mediated cleavage that removed the N-terminal MLS-HA-TEV sequence and associated proteins. Finally, the cell lysates or the resulting SelO protein complex was subjected to Western blotting using anti-SelO antibody under reducing and non-reducing conditions (Fig. 2C, D). One band (∼88 kDa) appeared only in the SXXC and CXXC samples under non-reducing conditions and the abundance of this 88 kDa protein complex was greater in the case of SXXC than the CXXC motif. This result strongly supported the existence of a potential redox target protein of SelO because the SXXC motif, in which the potential resolving Cys was replaced with Ser, enabled the mutant SelO to trap a redox target protein, whereas Ser substitution of the potential catalytic Cys (e.g., SXXS and CXXS motifs) prevented the formation of an interprotein disulfide bond. We further examined the effect of varying H_2_O_2_ concentrations and incubation times on the formation of the 88 kDa protein complex involving SXXC and SXXS mutant forms of SelO. The 88 kDa complex was detected for the SXXC form of SelO upon hydrogen peroxide treatment, but not in the case of SXXS (Fig. 3A). Moreover, the target protein disappeared with an increase in incubation times. These experiments provide evidence that human SelO interacts with a target protein in a redox manner, through the conserved CXXU motif. This interaction is reversible and depends on hydrogen peroxide treatment, representing a typical property of thiol-dependent oxidoreduction.

### Confirmation of Sec Incorporation into Full-length SelO

**Figure 4:**
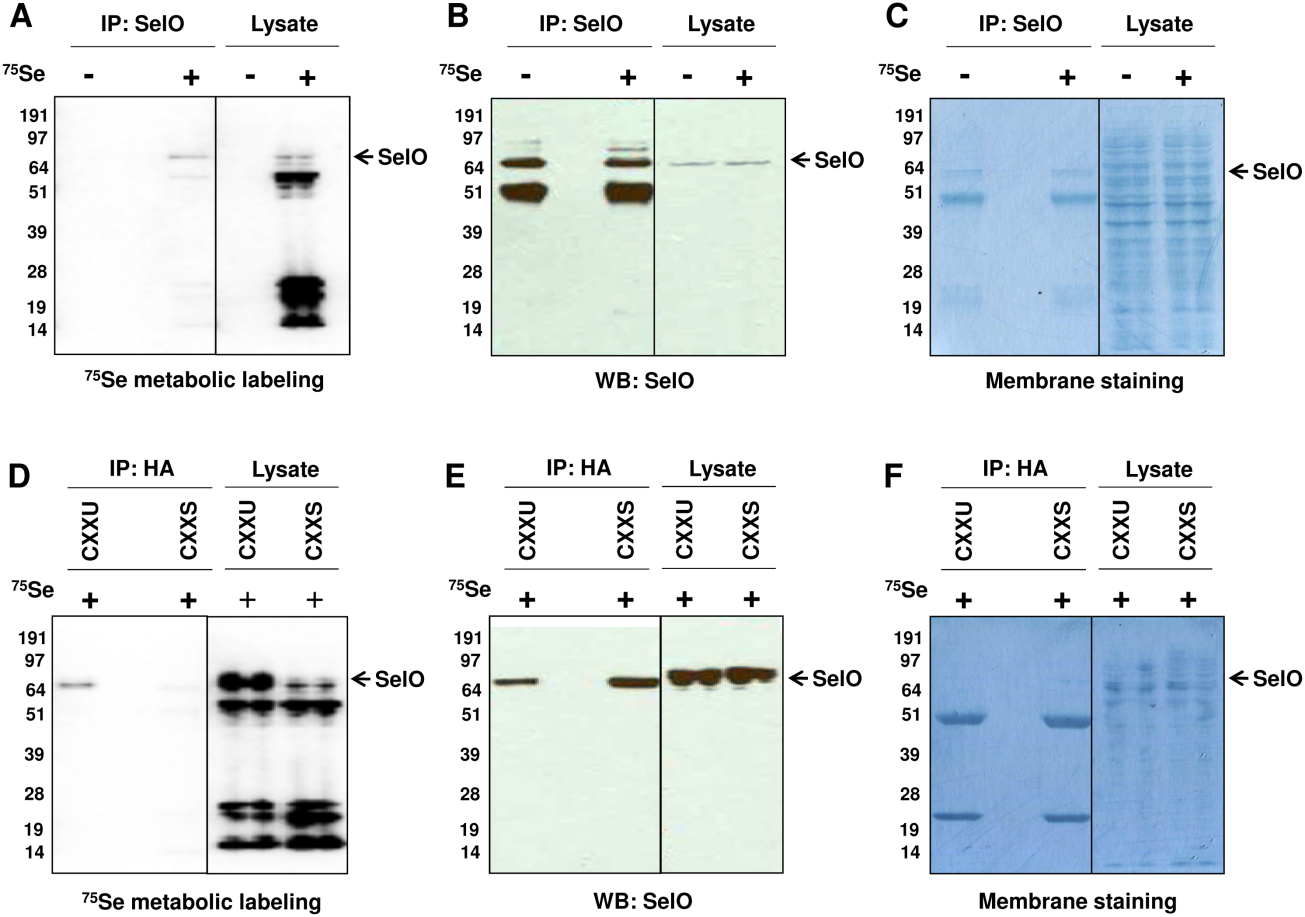
Incorporation of selenium into mammalian SelO. Endogenous SelO was metabolically labeled with ^75^Se in HEK293T cells. Cell lysate was immunoprecipitated with anti-SelO antibody and visualized by (A) phosphorimaging and (B) Western blotting with anti-HA antibody. (C) Amido black staining shows equal loading. The ^75^Se labeled cell lysate was also immunoprecipitated with anti-HA antibody. SelO in this sample was visualized by (D) phosphorimaging and (E) Western blotting with anti-SelO antibody. (F) Amido black solution was used to stain the membrane for loading control.

A study that discovered SelO as one of 25 mammalian selenoproteins also provided support for ^75^Se incorporation into these proteins, including a truncated form of SelO [6]. The full-length SelO, however, was not examined. Following ^75^Se metabolic labeling of HEK293T cells, SelO was immunoprecipitated with the SelO antibody and analyzed by phosphorimaging and Western blotting (Fig. 4A–C). The SelO band was detected by both methods, indicating that the endogenous SelO contains Sec. We further examined Sec incorporation into recombinant SelO proteins containing CXXU and the CXXS motifs. Following ^75^Se metabolic labeling, the recombinant SelO proteins were immunoprecipitated with the antibody to HA-tag and subjected to phosphorimaging and Western blotting. Both recombinant SelO forms were detected on Western blotting, but only the CXXU protein was detected by phosphorimaing (Fig. 4D–F). Taken together, these data show that the full-length SelO contains Sec in the C-terminal region.

### Regulation of SelO Expression by Dietary Selenium

**Figure 5:**
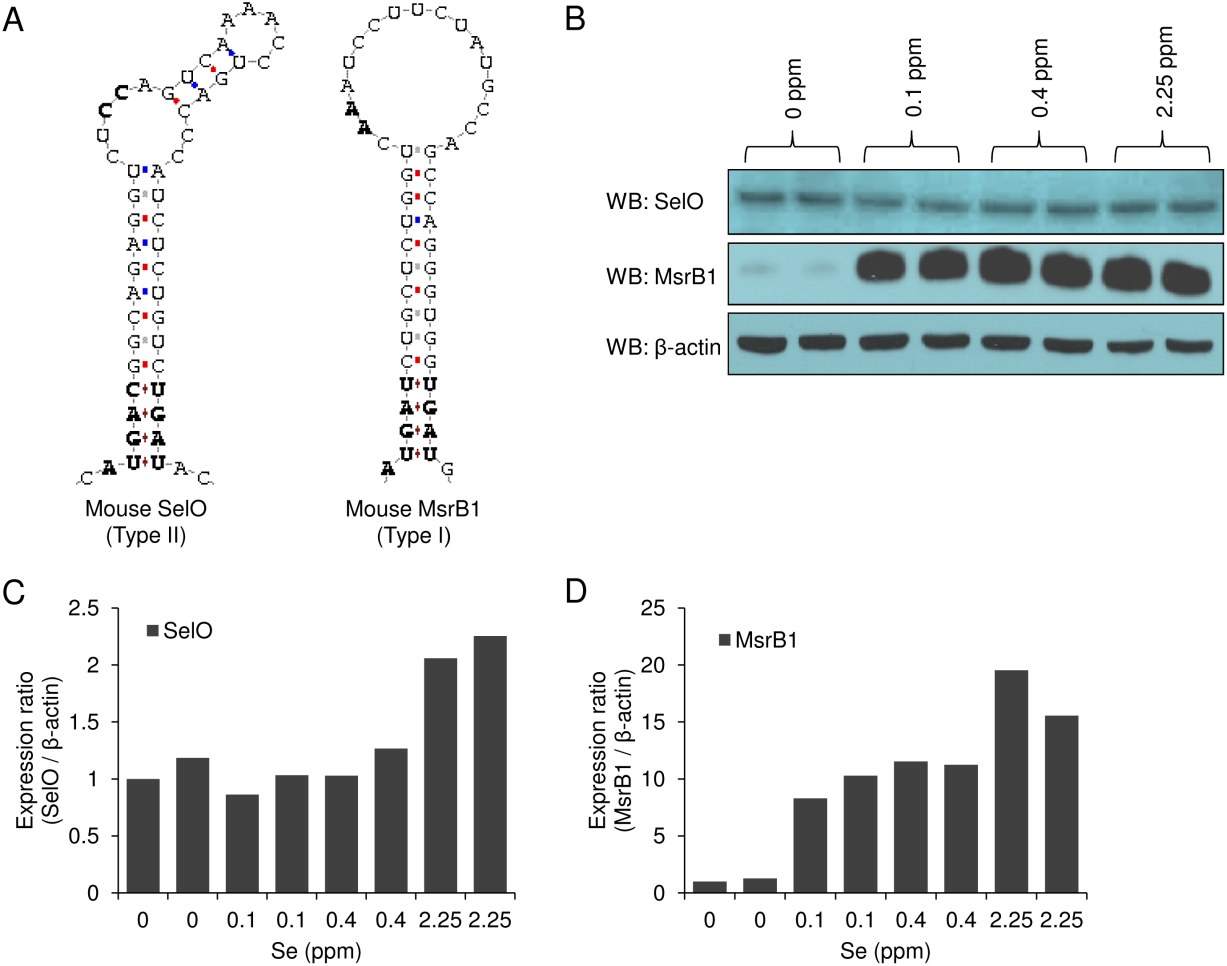
Regulation of SelO expression by dietary selenium. (A) Sec insertion sequence (SECIS) structure of mouse SelO and MsrB1. (B) Western blotting of SelO, MsrB1, and β-actin in liver lysates. Mice were fed various selenium diets (0, 0.1, 0.4, and 2.25 ppm) for 8 weeks. Expression ratio of (C) SelO to β-actin and (D) MsrB1 to β-actin. All quantifications were performed by using the Image J program.

Priority for Se supply in synthesizing selenoproteins is represented by hierarchy in selenoprotein expression upon selenium deficiency [24], [25]. For instance, dietary selenium deficiency dramatically reduces the expression of GPX1 and MsrB1 in mice, whereas the expression of TR1, TR2, and GPX4 is largely preserved under these conditions. Although the mechanism of this regulation is multifactorial and incompletely understood, reports point out the importance of the SECIS type in regulation of selenoprotein expression. For example, eIF4a3 was recently found to bind type 1 SECIS in GPX1 mRNA, whereas it did not interact with type 2 SECIS in GPX4 mRNA [24]. By using the SECISearch, we visualized SECIS structures in mouse SelO (COVE score: 22.22) and MsrB1 (COVE score: 23.75) (Fig. 5A). Interestingly, mouse SelO contains type 2 SECIS. We examined how the expression of SelO is regulated by dietary selenium. We carried out Western blotting of SelO, MsrB1, and β-actin in the liver lysates prepared from mice raised on various selenium diets (Fig. 5BCD). As expected, MsrB1 expression was dramatically reduced (i.e., more than 10 fold) by selenium deficiency diet (0?ppm). On the other hand, SelO expression under these conditions was not reduced as much (i.e., less than 2 fold). Thus, SelO can be classified as a selenoprotein with high priority for selenium supply.

It was recently hypothesized that SelO may have a kinase function, based on functional and structural prediction of its structure [19], [20]. These predictions require experimental verification. We demonstrate here that SelO is a mitochondrial selenoprotein engaged in redox interaction with an unknown protein through its CXXU motif. Redox regulation of protein function in mitochondria may involve kinase functions. For example, mitochondrial oxidation of ERK at Cys^38^ and Cys^214^ or AKT at Cys^60^ and Cys^310^ regulates kinase activity and signaling cascades [26]. It would be interesting to determine the identity of the SelO target protein, which may lead to direct functional insights for this protein.

## Supporting Information

Table S1 PCR primers used in cloning and preparation of chimeric gene expression constructs.
(DOCX)
