# Supplementary material for: Characterization of Mammalian Selenoprotein O: A Redox-Active Mitochondrial Protein
Source: PLoS One. 2014 Apr 21;9(4):e95518. doi: 10.1371/journal.pone.0095518 (PMC3994087; doi:10.1371/journal.pone.0095518)
Supplement: Table S1 — PCR primers used in cloning and preparation of chimeric gene expression constructs. (DOCX) [file pone.0095518.s001.docx]

**Supporting Information**

**Table S1. PCR primers used in cloning and preparation of chimeric gene constructs.**

| Primer | Sequence |
| --- | --- |
| 1. SelO MLS forward | 5’ - AATGCTAGCATGGCCGCATACAGGGCA - 3’ (NheI) |
| 2. SelO MLS reverse | 5’ - AATGAATTCCGGGGAACAGCGGCC - 3’ (EcoRI) |
| 3. SelO ORF forward | 5’ - AATTCTAGAATGTCGCCG GCGCCCCGC - 3’(XbaI) |
| 4. SelO ORF reverse (CxxU) | 5’ - ATGTCGACTTACGAAGATCATGTCACGCA - 3’ (SalI) |
| 5. SelO ORF reverse (CxxC) | 5’ - ATGTCGACTTACGAAGAGCATGTCACGCA - 3’ (SalI) |
| 6. SelO ORF reverse (CxxS) | 5’ - ATGTCGACTTACGAAGATGATGTCACGCA - 3’ (SalI) |
| 7. SelO ORF reverse (SxxU) | 5’ - ATGTCGACTTACGAAGATCATGTCACGCTCAGT - 3’ (SalI) |
| 8. SelO ORF reverse (SxxC) | 5’ - ATGTCGACTTACGAAGAGCATGTCACGCTCAGT - 3’ (SalI) |
| 9. SelO ORF reverse (SxxS) | 5’ - ATGTCGACTTACGAAGATGATGTCACGCTCAGT - 3’ (SalI) |
